# Circulating Tumour DNA Analysis for Tumour Genome Characterisation and Monitoring Disease Burden in Extramedullary Multiple Myeloma

**DOI:** 10.3390/ijms19071858

**Published:** 2018-06-24

**Authors:** Sridurga Mithraprabhu, Shreerang Sirdesai, Maoshan Chen, Tiffany Khong, Andrew Spencer

**Affiliations:** 1 Myeloma Research Group, Australian Centre for Blood Diseases, Alfred Hospital–Monash University, Melbourne 3004, Australia; durga.mithraprabhu@monash.edu (S.M.); maoshan.chen@monash.edu (M.C.); tiffany.khong@monash.edu (T.K.); 2 Malignant Haematology and Stem Cell Transplantation, Alfred Hospital, Melbourne 3004, Australia; s.sirdesai@alfred.org.au; 3 Department of Clinical Haematology, Monash University, Clayton 3800, Australia

**Keywords:** cell-free DNA, ctDNA, extramedullary MM, whole exome sequencing, spatial heterogeneity, digital PCR

## Abstract

Mutational characterisation in extramedullary multiple myeloma (EM-MM) patients is challenging due to inaccessible EM plasmacytomas, unsafe nature of multiple biopsies and the spatial and temporal genomic heterogeneity apparent in MM (Graphical abstract). Conventional monitoring of disease burden is through serum markers and PET-CT, however these modalities are sometimes inadequate (serum markers), not performed in a timely manner (PET-CT) and uninformative for identifying mutations driving disease progression. DNA released into the blood by tumour cells (ctDNA) contains the predominant clones derived from the multiple disease foci. Blood-derived ctDNA can, therefore, provide a holistic illustration of the major drivers of disease progression. In this report, the utility of ctDNA, as an adjunct to currently available modalities in EM-MM, is presented for a patient with EM and oligosecretory (OS) disease. Whole exome sequencing of contemporaneously acquired tumour tissue and matched ctDNA samples revealed the presence of spatial and temporal genetic heterogeneity and the identification of pathways associated with drug resistance. Longitudinal monitoring of plasma samples revealed that ctDNA can be utilised to define the dynamic clonal evolution co-existent with disease progression and as an adjunct non-invasive marker of tumour burden.

## 1. Introduction

Multiple Myeloma (MM) is a multi-focal genetically heterogeneous clonal plasma cell (PC) malignancy present at multiple intra-medullary sites within the bone marrow (BM). With disease progression, the PCs evolve the capacity to grow independently of the BM milieu and proliferate outside of the BM, manifesting as extramedullary (EM) MM and/or plasma cell leukaemia (PCL), with data showing that PCL represents a more genetically abnormal sub-clone that evolves from the original intra-medullary PC population [1]. Both spatial and temporal genetic heterogeneity are now recognised as adding to the genetic complexity of the disease as it evolves [2,3,4,5,6]. The incidence of EM soft-tissue plasmacytomas in patients at diagnosis is 7% with an incidence of almost 20% at the time of relapse [7]. Clinical outcome in patients with EM-MM is associated with an adverse prognosis, with <6 months median overall survival for relapsed patients and the clinical management of patients being particularly challenging [8,9,10,11]. Conventional markers of tumour burden (paraprotein (PP) and serum-free light chains (SFLC)), BM biopsy and whole-body PET/CT are currently the accepted modalities for measuring tumour response in EM-MM patients. Despite the availability of these standard diagnostics for monitoring of disease burden there remains a critical need for newer strategies as it is now recognised that single site biopsies fail to capture the spatial and temporal genetic complexity of this multi-focal disease [3,4,12]. This is exemplified by the observation that spatiotemporal comparisons of BM and targeted biopsies of EM disease has indicated that subclones absent from the BM may be present at EM sites and that these subclones can respond differentially to treatment [3,4,5]. While this targeted biopsy approach has confirmed spatiotemporal intraclonal heterogeneity, it is invasive, may be subject to sampling bias and not always feasible due to the location of EM plasmacytomas. Additionally, conventional methodologies cannot determine the underlying driver mutation of disease progression in patients with differential response to treatment. Therefore, a more practical approach to determining the driver mutations and to evaluate treatment response is required. An alternative validated approach is the use of circulating cell-free tumour derived DNA (ctDNA). Tumour cells shed small amounts of nucleic acids into the blood stream and ctDNA from peripheral blood plasma (PB-PL) theoretically contains a representation of the entire multi-focal tumour genome. Accumulating data suggests that the interrogation of PL-ctDNA may have clinical utility and could be a valuable diagnostic addition to conventional markers of tumour burden [13,14,15,16]. This becomes specifically relevant for patients with oligosecretory (OS) or non-secretory (NS) MM and in patients with EM-MM disease, wherein conventional markers of tumour burden are not adequate and biopsy of the site is always not plausible, respectively. Whole exome sequencing (WES) of ctDNA for characterisation of the mutational spectrum enables evaluation of mechanisms of tumour progression or treatment resistance [17,18,19,20]. In this study, we present a case report of a patient with OS- and EM-MM and the utility of ctDNA for mutational characterisation and as a marker of tumour burden in the absence of BM disease and persisting EM plasmacytomas.

## 2. Results

### 2.1. Case Description

A 58-year old lady was diagnosed with OS kappa light chain (KLC) myeloma in June 2014 after a history of worsening back pain over the preceding four months. At admission, a CT scan showed multi-level crush fractures at T8, T9, L1 and L2; (a sestamibi scan later confirmed avid uptake in the axial skeleton). She was hypercalcaemic at presentation (Ca^2+^ corrected 3.33 mmol/L), with mild renal impairment (Creatinine 133 μmol/L) and a haemoglobin of 110 g/L. Circulating plasma cells (PC) were seen (22% of 10.1 × 10^9^/L total white cells) on the peripheral blood film, consistent with PCL. No PP was detectable and KLC were only modestly elevated at 112 mg/L. The LDH and albumin were normal and beta-2-microglobulin (B2M) was elevated at 4.4 mg/L. A BM biopsy was diagnostic of MM with a marrow burden of 80% PC infiltration. FISH studies subsequently demonstrated a 17p deletion.

The patient was treated with 4 cycles of Bortezomib/Cyclophsophamide/Dexamethasone (VCd) followed by a melphalan (200 mg/m^2^) conditioned autograft in November 2014. This resulted in a modest reduction in BM PC burden from 80% to 20%. Given the high-risk MM features and the availability of a HLA matched unrelated donor, the patient underwent a fludarabine/TBI (2 Gy) conditioned allograft in February 2015. Cyclosporin and mycophenolate were used for post-transplant graft verus host disease (GvHD) prophylaxis. 

No meaningful disease control was achieved post-allograft. At day 42, KLC rose to 641.2 mg/L. As there were no features of GVHD, a rapid wean of her immunosuppression was instituted. The patient had mixed CD3 chimerism at day +60 with 79% being donor derived. The reduction in immunosuppression temporarily halted the progressive increase in KLC but at the cost of mild hepatic and cutaneous GvHD. Low level systemic immunosuppression was thus reintroduced with a corresponding rebound in KLC. A BM biopsy at 6 months post allograft in August 2015 showed extensive PC infiltration and the KLC had increased to 840 mg/L. At this point, immunosuppression was weaned again and thalidomide (T) 100 mg daily was commenced. The patient remained symptomatically well.

**Figure 1:**
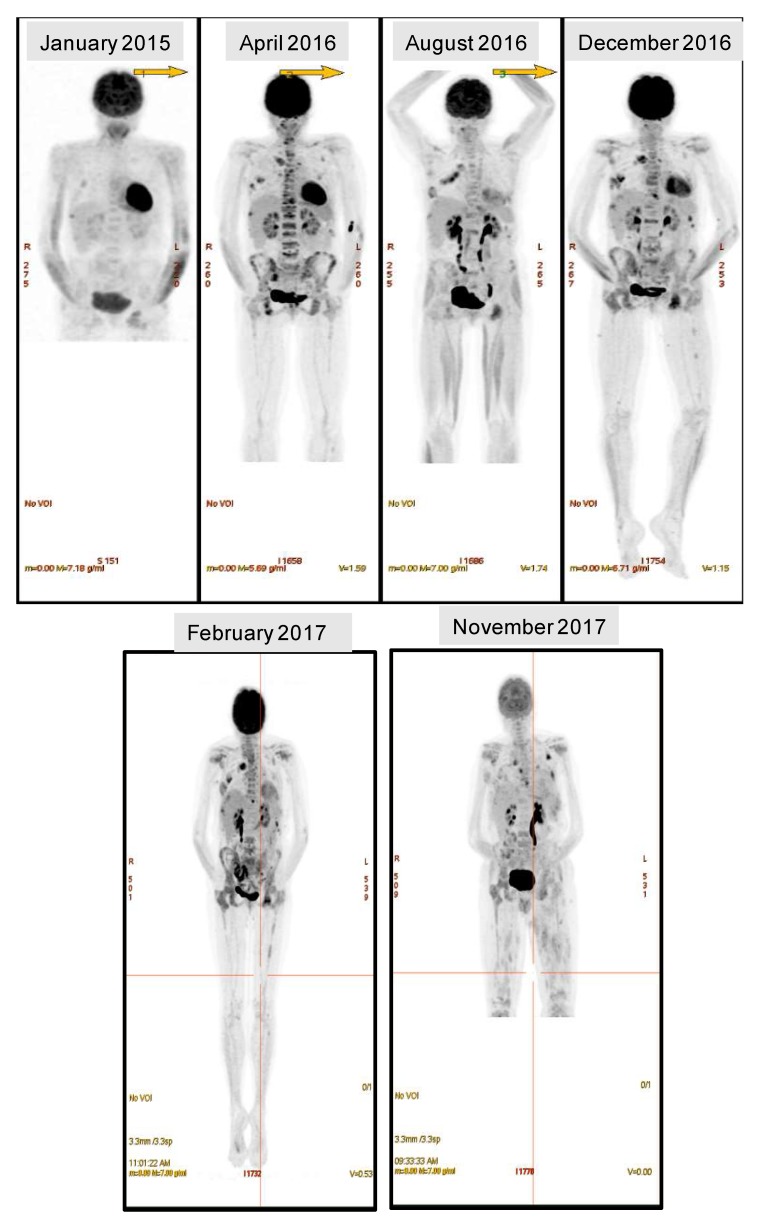
Progression of disease. Positron Emission Tomography (PET) images indicate the progression of disease in the extramedullary (EM)-sites with relative response to treatment in the bone marrow (BM).

The combination of T and the withdrawal of immunosuppression led to a reduction in KLC to 148 mg/L in February 2016, 1 year post allograft, though moderate BM involvement persisted. CD3 chimerism had improved to 100% by day +180 and remained complete at 12 months. However, despite ongoing biochemical control (KLC 150 mg/L), the patient presented to hospital with a pathological clavicular fracture in April 2016. A PET-CT confirmed widespread skeletal disease and the patient was switched to lenalidomide (R) (Figure 1). She presented again in late July 2017 with progressive lower limb weakness. MRI demonstrated T12 spinal cord compression from extra-osseous disease. A repeat PET-CT showed differential response of the widespread FDG-avid lesions since the commencement of the R (Figure 1). KLC remained suppressed −165.8 mg/L. She was treated with radiotherapy and dexamethasone (d) and regained full mobility with continuation of R treatment.

Biochemical control was maintained on Rd until November 2016 when she presented with new thoracic wall disease and by early mid-December 2016, KLC had risen to 347 mg/L. PET-CT scan showed clear progression with numerous subcutaneous deposits (Figure 1). The patient became cytopenic and a repeat BM biopsy demonstrated effacement of normal haemopoiesis by PC. Both the BM and EM-sternum (EM-S) biopsy were subject to genomic analyses along with a matched PB sample (315) for ctDNA assessment. In an attempt to systemically target the EM disease, she was commenced on a combination of panobinostat, bortezomib and Dex (PAN-Vd), but this failed to arrest disease progression. By February 2017, KLC were 1200 mg/L despite modest reductions in the size of some cutaneous plasmacytomas. Salvage treatment with pomalidomide (P), C and d (PCd) was commenced. This led to a slight, reduction in light chains (1200 mg/L to 600 mg/L) but the EM disease progressed and she experienced further spinal cord compression in April 2017. This was again treated with palliative radiotherapy. Over the next few months she required radiotherapy to multiple cutaneous plasmacytomas.

In May and July 2017, the patient received two donor lymphocyte infusions (DLI). Dexamethasone was withheld but P was continued during this period. The DLIs caused a fleeting reduction in KLC, but, again the patient had EM progression and presented with further spinal cord compression in July 2017, again requiring further palliative radiotherapy. Additional cfDNA samples were obtained in July and September 2017 (435 and 485). Carfilzomib (K) and daratumumab (Dara) were accessed and combined with Pd (KPd-Dara); though the quadruplet achieved a degree of biochemical control (nadir 107 mg/L, late September 2017), the patient’s EM disease remained resistant to treatment with PET-CT showing clear progression with numerous subcutaneous deposits in November 2017. Biopsy from 3 different regions of an axillary nodal EM-plasmacytoma (EM1, 2 and 3) were obtained along with a matched cfDNA sample (504). She presented again in December 2017 with cord compression; further radiotherapy was delivered but the patient deteriorated, becoming increasingly obtunded. She was palliated and died shortly thereafter.

### 2.2. Spatial Genomic Heterogeneity in EM-MM

**Figure 2:**
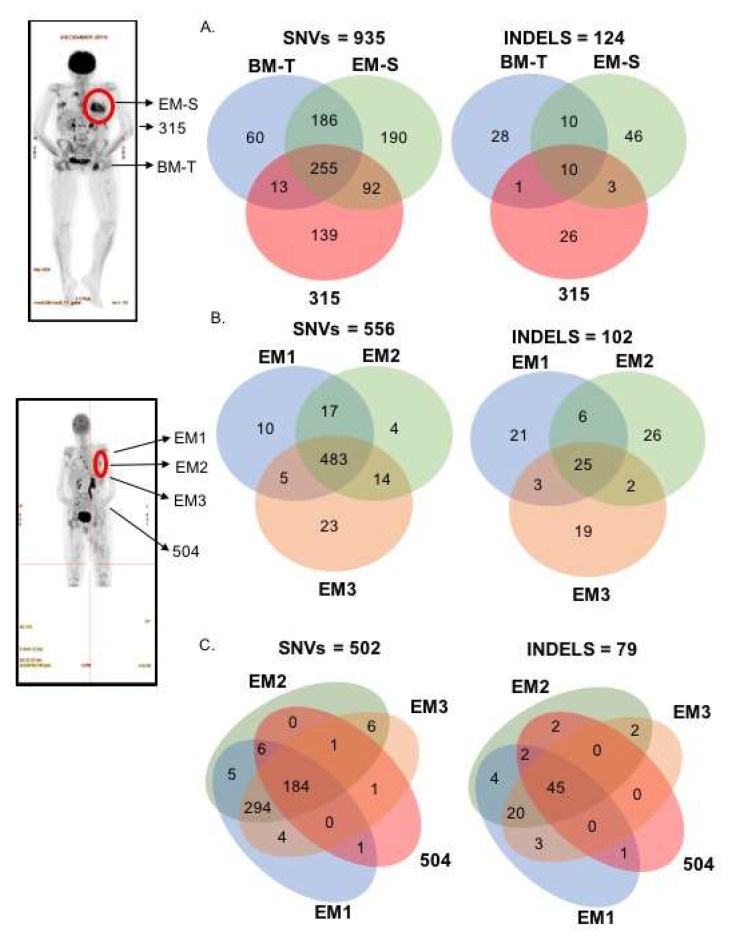
Spatial genetic heterogeneity. Spatial genomic heterogeneity was evident in comparisons of EM-tissues, BM biopsy and ctDNA (**A**) Left and right venn diagrams indicates the presence of common and exclusive SNV and INDELS, respectively, in time-matched EM-S, BM-T and 315 (**B**) Left and right venn diagrams indicates the presence of common and exclusive SNV and INDELS, respectively, in an axillary EM-plasmacytoma biopsied at three locations (EM1, 2 and 3) (**C**) Left and right venn diagrams indicates the presence of common and exclusive SNV and INDELS, respectively, in time-matched EM-plasmacytoma biopsied at three locations (EM1, 2 and 3) and ctDNA sample 504. Red circles on PET image indicate the areas of EM-tissue biopsy.

EM-biopsy samples at two time points (November 2016 (EM-S) and 2017 (EM1, 2 and 3)), BM-trephine (BM-T; matched to November 2016) and sequential PL ctDNA from four time points (November 2016 (315), July (435), October (485) and November 2017 (504)) were obtained from the patient and subject to WES to identify single nucleotide variants (SNVs) and INDELS (Figure 2). Multi-site spatial heterogeneity in time-matched samples were initially assessed. EM-S, BM-T and 315 were observed to harbour 935 SNVs, of which, 27% (255) were present in all three samples, 31% (291) in two and 42% (389) were found at only 1 site (Figure 2A, left venn diagram). Additionally, 27% of the SNVs was shared between BM-T and 315, while 37% of SNVs were shared between EM-S and 315. Within the 124 INDELS identified, 81% (100) were unique and found in only 1 site, 11% (14) were found in 2 sites and only 8% were present in all three sites (Figure 2A, right venn diagram). To demonstrate spatial heterogeneity within single foci, 3 locations from a single axillary EM-plasmacytoma site in November 2017 were obtained (EM 1, 2 and 3). Analyses revealed that of a total of 556 SNVs, 483 were common to all three locations, with 36 SNVs present in only 2 and 37 SNVs in only 1 site (Figure 2B). In contrast, only 25 INDELS were present in all 3 sites, with a majority of the INDELS (66/102) present in only 1 site (Figure 2B). Time-matched multi-site comparison was also plausible for the EM1/2/3 and 504 samples and all SNVs and INDELS that were present in two or more samples were plotted. Comparison of intra-tumoural heterogeneity and spatial heterogeneity revealed that only 37% (184/502) SNVs and 57% (45/79) of INDELS were present in all samples (Figure 2C).

### 2.3. Temporal Heterogeneity and Biology of Disease Progression

Temporal genetic heterogeneity was assessed using EM-S vs EM1/2/3 to determine genomic changes in tissue samples during disease progression. All SNVs and INDELS detected in the EM-S plasmacytoma was detected in the EM1/2/3, with the later tissue sample acquiring 67 other SNVs and 16 INDELS, notably, NRAS Q61H (gain-of-function), FGFR3 I245N (loss of function) and a frameshift deletion of the ANGPTL7 exonic region/MTOR intronic gene (Q122fs*23) (Supplemental File S1). Pathway enrichment analysis also indicated that EM1/2/3 had significantly enriched variants in the “Cancer Drug Resistance by Drug Efflux”, “Myc Mediated Apoptosis Signalling” and “PI3K signalling in B Lymphocytes” pathways, amongst others (Supplemental File S1). Disease evolution was also analysed utilising the ctDNA samples to ascertain the mechanisms of drug resistance. The top 5 significantly enriched pathways between these four samples were assessed to reveal that “Protein Ubiquitination Pathway” was amongst the top 2 in all samples (Table 1, Supplemental File S1). Two other pathways that were significantly enriched in the final cfDNA sample (504) were “Protein Kinase A Signalling” (PKA; *p* = 0.0001) and “Wnt/β-catenin Signalling” (*p* = 0.003). While the PKA pathway was identified in other three ctDNA samples, although not within the top 5, the Wnt/β-catenin Signalling pathway was unique to 504.

**Figure 3:**
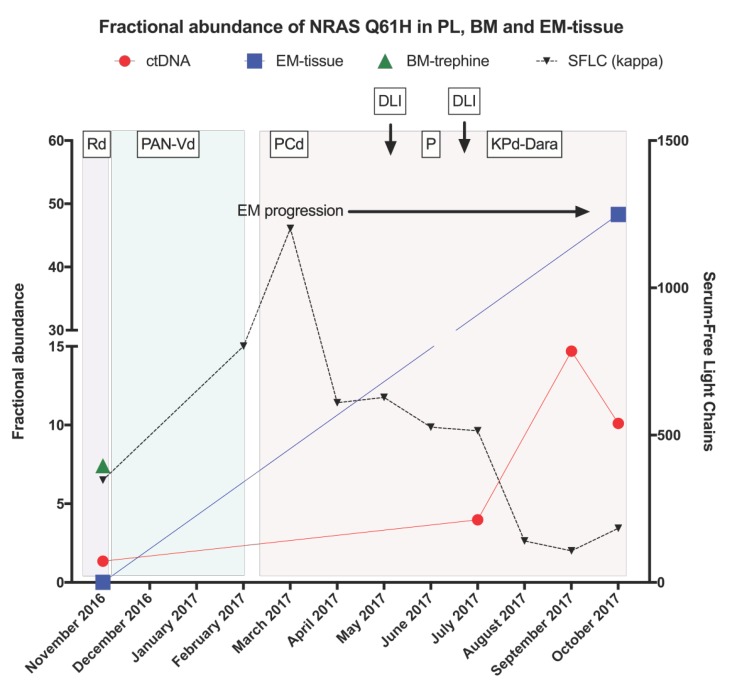
Longitudinal tracking of NRAS Q61H mutation as an evidence for spatial heterogeneity and as a marker of tumour burden. FA of NRAS Q61H in sequential PL, BM-trephine and EM tissues. EM-S obtained in November 2016 did not have any detectable levels of NRAS Q61H, however, this is a dominant clone in EM1/2/3 obtained in November 2017. BM-T collected in November 2016 did demonstrate NRAS Q61H, however, BM disease subsequently responded to treatment but with persisting disease in the EM sites. KLC show an increase until March 2017 and reduction with the introduction of PCd. Single agent P was continued until mid-June 2017 when carfilzomib (K) and daratumamab (dara) were added. The ctDNA levels increase steadily across a period of 12 months, in spite of the decreasing KLC levels.

A validation of spatial and temporal heterogeneity was undertaken using ddPCR for the NRAS Q61H mutation. This mutation was detected by WES on BM-T and in EM1/2/3, and undetectable in ctDNA or EM-S. However, when ddPCR was performed it was observed that all ctDNA, BM-T and the EM1/2/3 samples expressed the mutation, but it still remained undetectable in the EM-S tissue. This confirms the presence of temporal heterogeneity between the two EM-plasmacytomas. Furthermore, as the sensitivity of ddPCR is relatively higher than WES (0.01% vs. 5%, respectively), the presence of NRAS Q61H mutation in the ctDNA was discovered, indicating that this methodology can be particularly useful for validating mutations that may be undetectable by WES (Figure 3).

### 2.4. ctDNA Analysis for Disease Progression

**Figure 4:**
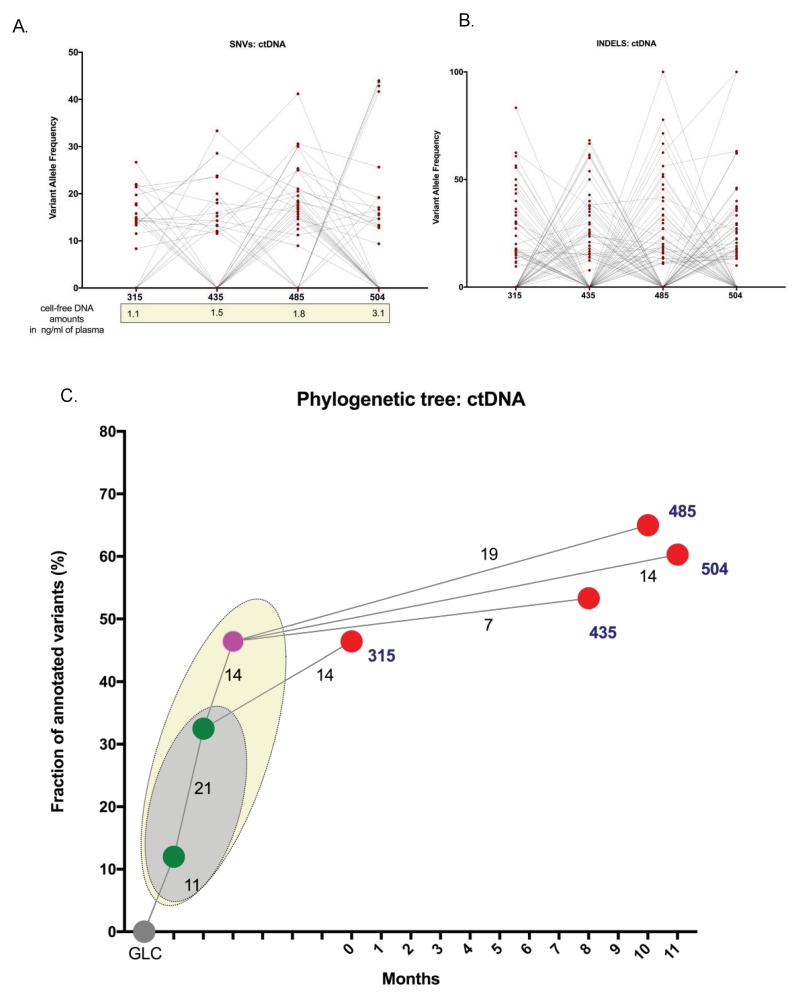
Clonal evolution using ctDNA. The VAFs of SNVs and INDELS were compared across the four ctDNA sampling time points. (**A**) All plotted SNVs had a COSMIC database annotation. The disappearance and emergence of the different subclones are evident and indicates the changing disease kinetics. The amount of cell-free DNA present in each sample is also indicated. (**B**) The VAFs of all INDELS identified in ctDNA samples also indicating the presence of INDELS at all time points coincident with the presence of disease in the patient. (**C**) Phylogenetic tree depicting evolutionary distances between sequential ctDNA specimens 315, 435, 485 and 504, depicted as collected at month 0, 8, 10 and 11, respectively. Evolutionary distance is defined as the fraction of common and uncommon mutations shared between the four ctDNA samples. The grey node indicates the germline (GLC). All samples have shared mutations, present in all 4 or 3 (green nodes, including the earliest sample 315), indicated by the grey oval. The pink node, represents common mutations that were shared 435, 485 and/or 504 and not found in 315, and is derived from the ancestral shared clones (shown by the light-yellow oval). The red nodes indicate the divergent clonal evolution that is private to the specific samples. The length of the individual branches denotes the proportion of mutations separating the two disease events.

PET-CT images from February 2017 and November 2017 indicate that the disease was responding at the intra-medullary sites, however, there were a number of persisting EM plasmacytomas (Figure 1). The fractional abundance (FA) of NRAS Q61H was measured in ctDNA obtained at 4 time points between November 2016 and November 2017. The levels of NRAS Q61H in PL continued to rise in the context of persisting EM disease, showing refractoriness to three different P-containing regimens, in contrast to the KLC that demonstrated a paradoxical serological response. Moreover, the absence of serological progression between February 2017 and November of 2017, was inconsistent with the ctDNA NRAS Q61H FA demonstrating that the ctDNA levels were a more informative marker of disease progression (Figure 3). Furthermore, a panel of SNVs with a COSMIC database annotation, identified by WES in the ctDNA samples, was plotted with the observed variant allele frequencies (VAF) to determine whether evolution could be correlated with disease status (Figure 4A, Supplemental File S1). There were no significant differences between the VAFs across the time points, however, the emergence and disappearance of a number of clones was observed within all ctDNA samples indicating the presence of different but persisting clones driving the progression of the disease over time. Likewise, all INDELS detected in the ctDNA were plotted to demonstrate the clonal evolution and disease progression (Figure 4B). A comparison of the evolutionary history of the sequential ctDNA by plotting the fraction of common and uncommon annotated SNVs was also performed (Figure 4C). A phylogenetic tree was derived depicting evolutionary distances between the ctDNA samples using a previously described convention [21,22,23]. Although a number of common mutations were evident in all four ctDNA samples, persisting disease and dynamic alterations in the tumour genome resulted in the acquisition of phase-specific mutations exclusive to the sample from an ancestral clone.

## 3. Discussion

In this report, the utility of ctDNA to be used as a non-invasive quantitative (tumour burden) and qualitative (characterisation of the tumour genome) biomarker in MM patients, focusing on an EM-MM patient with high risk OS disease wherein standard methodologies were inadequate, was undertaken. Mutational characterisation in the EM-MM is challenging due to inaccessible plasmacytomas and/or unsafe nature of performing multiple biopsies to obtain tissue for characterisation. This is particularly important, as previously published literature indicates that spatiotemporal heterogeneity is apparent between the disease foci using a multi-site biopsy approach [2,4,12]. We have previously demonstrated that spatial heterogeneity could be identified with the use of ctDNA utilising a pre-designed mutation panel [14]. This report provides a more comprehensive, albeit less sensitive approach, WES to demonstrate heterogeneity. Samples sourced from multiple sites and multiple locations within a single EM-plasmacytoma site has demonstrated both spatial and temporal heterogeneity and for the first time intra-tumoral heterogeneity. While a number of common SNVs and INDELS were identified in all samples, there were clear differences between the samples collected during the course of disease progression. This concept was validated by ddPCR through assessment of NRAS Q61H, wherein, despite the ctDNA NRAS Q61H being undetectable with WES, the presence of NRAS Q61H in all samples except EM-S at a subclonal level, was confirmed by ddPCR, demonstrating that ctDNA can harbour mutations that originate from all sites. The BM/EM-MM samples contain enriched ‘tumor specific’ DNA while the PL cfDNA pool consists of both normal and (multi-site) tumour DNA. Therefore, it can be stated with a high degree of confidence that mutations detected only at the biopsy site but not represented in the PL analysis may be ‘locally’ predominant but are not representative of the predominating clones in the overall tumour burden within this patient.

The comparison of SNVs and INDELS in ctDNA samples and in EM-tissues has enabled the identification of pathways involved in disease progression in this patient. Within the EM-tissues, EM1/2/3 tissue had a significant enrichment of pathways that were not identified in EM-S indicating that the disease had evolved with time. Similarly, pathway analysis in the ctDNA samples, while indicating the presence of disease, also provided evidence for the dynamic disease kinetics that could be captured with ctDNA analysis. Importantly, some of the pathways identified in the ctDNA could be therapeutically targeted, for instance, the Wnt/β-catenin Signalling [24], providing a platform for precision medicine. Additionally, it also provides a discovery platform of mutations/pathways driving disease progression, not previously identified in MM.

Longitudinal tracking of specific mutations in PL ctDNA in patients undergoing therapy has indicated the ctDNA provides a more holistic marker of tumour burden compared to conventional markers. This was demonstrated by the presence of NRAS Q61H in the PL ctDNA coincident with disease progression but falling KLC. This observation was further validated with the visualisation of the fluctuating landscape of VAFs of SNVs and INDELS in the ctDNA during disease course, providing evidence for both presence of tumour burden and the clonal dynamics concurrent with drug resistance. Together, these results indicate that WES facilitates comprehensive clonal analysis of cfDNA to track tumour burden, evolution and for identification of mechanisms of drug resistance in MM patients and will be particularly useful in patients with inadequate disease monitoring methodologies. Additionally, ctDNA analysis enables significantly improved diagnostic capabilities via a non-invasive complication-free approach thus informing personalised treatment approaches with exciting implications for our understanding of the biology of MM.

## 4. Materials and Methods

### 4.1. Peripheral Blood (PB) Collection and Processing

Peripheral blood plasma (PL) in Streck BCT DNA (Streck, La Vista, NE, USA) were collected at four timepoints (November 2016, July, October and November 2017) following informed consent. Immediately upon sample collection, the tubes were inverted to mix the blood with the preservative in the collection tube. PL was separated from PB through centrifugation at 820 g for 10 minutes (min) within 24 h of sample collection. Supernatant was collected without disturbing the cellular layer and centrifuged again at 16,000× g for 10 min to remove any residual cellular debris and stored at −80 °C in 1 mL aliquots for long-term storage until isolation. PB from EDTA tubes were subjected to ficoll isolation of peripheral blood mononuclear cells (PBMC) respectively, to be utilised as germ line controls for WES analyses. PBMC were snap frozen as cell pellets and stored at −80 °C until DNA extraction.

### 4.2. Cell-Free DNA Extraction

Frozen PL samples were used for cfDNA extraction using the QIAamp circulating nucleic acid kit (Qiagen, Hilden, Germany) according to manufacturers’ instructions. Approximately, 4 mL of PL was used for extractions and the cfDNA was eluted in 100 µL of buffer AVE. Subsequently, PL cfDNA was quantified with a QUBIT Fluorometer 3.0 and high sensitivity DNA detection kits (Thermo Fisher Scientific, Waltham, MA, USA). The maximum input volume utilised for the QUBIT assay was 5 µL. The extracted cfDNA were stored at −80 °C until further processing.

### 4.3. Genomic DNA Extraction from PBMC/EM Tissue Biopsy/Trephine

Biopsy of the EM tissue at two time points were collected (November 2016 and 2017) with a single sample of the sternum site and three spots from an axillary site, respectively (Figure 2). Extraction of DNA from PBMC and EM tissues were performed using the QIAGEN Blood DNeasy Kit [14]. BM trephine was collected at November 2016 and was utilised for extraction of DNA utilising the High Pure FFPET DNA isolation kit (Roche, Basel, Switzerland). Briefly, a single slice of 10 micron sections of formalin fixed paraffin-embedded (FFPE) BM trephine was utilised for DNA extraction and all procedures according to instructions were followed. Subsequently, DNA was quantified with a QUBIT Fluorometer 3.0 and high sensitivity DNA detection kits, as before.

### 4.4. Whole Exome Sequencing and Identification of Somatic Variants

Genomic, trephine and ctDNA DNA were utilised for library preparation. Between 400 and 600 ng of input DNA was utilised for EM1/2/3 and BM-T and between 20 and 100 ng DNA was utilised for ctDNA and EM-S samples (Supplemental File S1). Sequencing libraries were generated using Agilent V6 Exome capture kit (Agilent Technologies, Santa Clara, CA, USA) following manufacturer’s recommendations and x index codes were added to attribute sequences to each sample. Briefly, fragmentation was carried out by hydrodynamic shearing system (Covaris, Woburn, MA, USA) to generate 180–280 bp fragments. Remaining overhangs were converted into blunt ends via exonuclease/polymerase activities and enzymes were removed. After adenylation of 3′ ends of DNA fragments, adapter oligonucleotides were ligated. DNA fragments with ligated adapter molecules on both ends were selectively enriched in a PCR reaction. Captured libraries were enriched in a PCR reaction to add index tags to prepare for hybridization. Products were purified using AMPure XP system (Beckman Coulter, Beverly, MA, USA) and quantified using the Agilent high sensitivity DNA assay on the Agilent Bioanalyzer 2100 system. Sequencing was performed using 150 bp paired-end sequencing on Illumina HiSeq4000. The average sequencing depth for both ctDNA and tissues was 100×. Generated data was aligned to the reference genome using the Burrows-Wheeler Aligner (BWA V0.7.16a, http://bio-bwa.sourceforge.net/), processed through Picard (http://broadinstitue.rithub.io/picard/) and the GATK pipeline. Ingenuity variant analysis (IVA) software was utilised for variant annotation and pathway enrichment analysis. SNVs with a depth of coverage <20 in tumour or PL samples and failed upstream filtering were excluded. Variants outside top 5.0% most exonically variable 100 base windows in healthy public genomes (1000 genomes) and outside top 1.0% most exonically variable genes in healthy public genomes (1000 genomes) were included. Excluded variants were ones observed with an allele frequency greater than or equal to 3.0% of the genomes in the 1000 genomes project OR greater than or equal to 3.0% of the NHLBI ESP exomes (All) OR greater than or equal to 3.0% of the AFC Frequency OR greater than or equal to 3.0% of the ExAC Frequency OR greater than or equal to 3.0% of the gnomAD Frequency OR Filter variants unless it was an established pathogenic common variant. The default filter settings on IVA for ‘predicted deleterious’ and ‘cancer driver variants’ were employed. SNVs and INDELS appearing in the germ line control were excluded utilising the ‘tumour-specific variants’ setting. Phylogenetic tree with the evolutionary pattern distinguishing the different PL samples were derived using previously described convention based on defining the fraction of common and uncommon mutations in each sample [21,22,23].

### 4.5. Droplet Digital PCR

All samples were diluted to the same amount for ddPCR and atleast 5 ng of DNA was added for each sample, including the positive and negative control samples. Samples from patients were quantitatively tracked using ddPCR Prime PCR assay conditions (Biorad, CA, USA; QX200 droplet digital PCR system). All samples were run in quadruples for detection of target mutation. A minimum of three positive droplets across the four wells was required for a positive result for detection of rare events. Following PCR, the droplets were read with a two-fluorescence detector to determine droplets that were positive for the mutation of interest. Quantasoft™ software version 1.7 (Bio-Rad, Hercules, CA, USA) enabled the determination of the number of mutant copies and fractional abundance (FA) of the samples. The “Ratio” tab in the Quantasoft™ software provides the FA of the sample, which is a ratio of the *Mutation/(Wildtype + Mutation)*. The fractional abundance of mutant copies relative to wild type copies using the “merged wells” setting was recorded, which takes into account droplets across the four replicates of each sample. The FA values of each of the samples including the positive and negative controls has been provided as Supplemental Figure S1.

## Figures and Tables

**Table 1:** List of top 5 significantly enriched pathways in ctDNA. Pathway enrichment analysis was performed for the SNVs detected in each of the ctDNA samples to determine significantly enriched pathways. The top 5 pathways in each ctDNA sample is listed.

315	435	485	504
Protein Ubiquitination Pathway	Glutamate Receptor Signalling	Protein Ubiquitination Pathway	Protein Kinase A Signalling
Aldosterone Signalling in Epithelial Cells	Protein Ubiquitination Pathway	Epithelial Adherens Junction Signalling	Protein Ubiquitination Pathway
Unfolded protein response	Estrogen Receptor Signalling	Th1 and Th2 Activation Pathway	Huntington’s Disease Signalling
Huntington’s Disease Signalling	Interferon Signalling	Germ Cell-Sertoli Cell Junction Signalling	nNOS Signalling in Neurons
Role of Macrophages, Fibroblasts and Endothelial Cells in Rheumatoid Arthritis	Corticotropin Releasing Hormone Signalling	Th1 Pathway	Wnt/β-catenin Signalling

## Supplementary Materials

Supplementary materials can be found at http://www.mdpi.com/1422-0067/19/7/1858/s1.
