# Supplementary figures and images for: Circulating Tumour DNA Analysis for Tumour Genome Characterisation and Monitoring Disease Burden in Extramedullary Multiple Myeloma
Source: Int J Mol Sci. 2018 Jun 24;19(7):1858. doi: 10.3390/ijms19071858 (PMC6073672; doi:10.3390/ijms19071858)

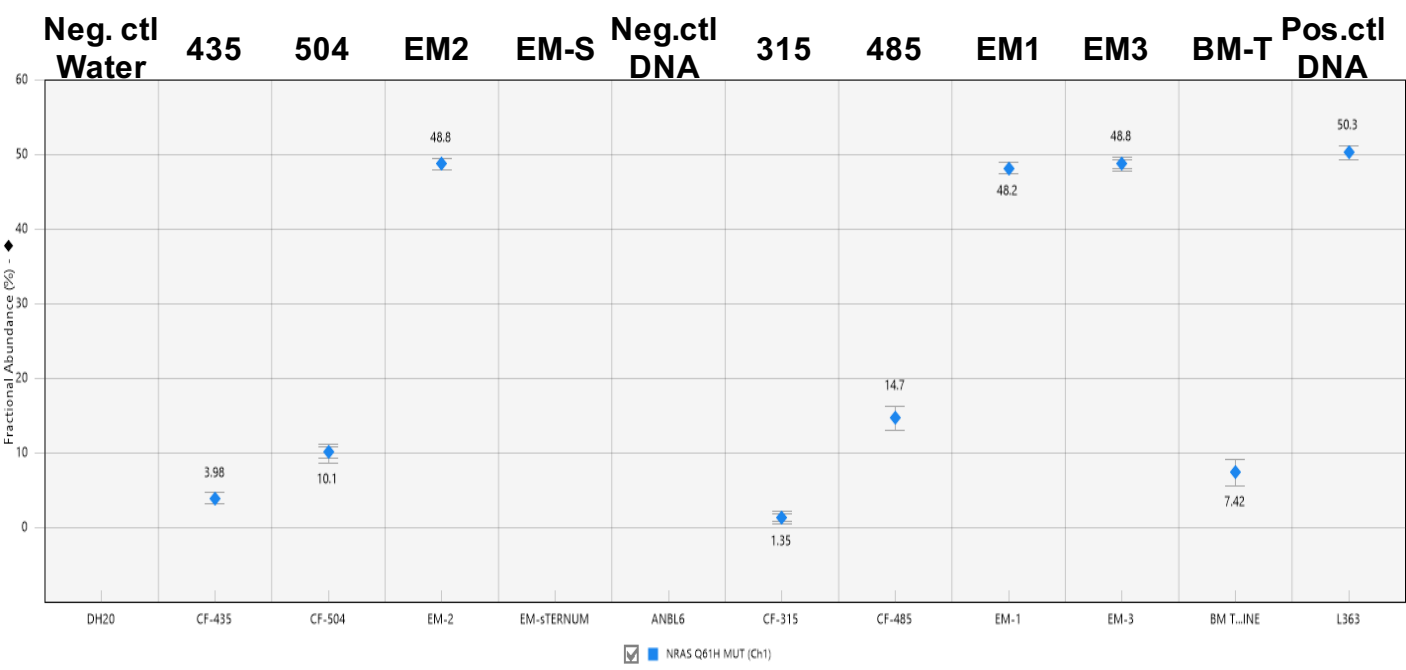

Supplement: Supplementary file 1 [file ijms-19-01858-s001.zip › ijms-320037-supp/Supplemental Figure S1.pdf]
